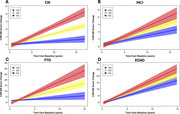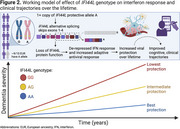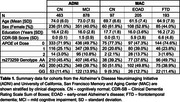# A common *IFI44L* variant strongly predicts clinical trajectories in aging and neurodegeneration

**DOI:** 10.1002/alz70855_104509

**Published:** 2025-12-24

**Authors:** Luke W. Bonham, Alexis P. Oddi, Valerie Drews Escobar, Jared A. Khan, Ariel I. Sauri, Patricia A. Castruita, Taylor P. Johnson, Yaen Chen, Robert Loughnan, Xin Wang, Gisele Sanda, Phuong T. Hoang, Leo P. Sugrue, Ole A. Andreassen, Anders M. Dale, Michael J. Corley, Gil D. Rabinovici, David C. Perry, William W. Seeley, Bruce L. Miller, Iris J. Broce, Daniel W. Sirkis, Jennifer S. Yokoyama

**Affiliations:** ^1^ Department of Radiology and Biomedical Imaging, University of California, San Francisco, San Francisco, CA, USA; ^2^ Memory and Aging Center, Department of Neurology, Weill Institute for Neurosciences, University of California, San Francisco, San Francisco, CA, USA; ^3^ Graduate Program in Pharmaceutical Sciences and Pharmacogenomics, University of California, San Francisco, San Francisco, CA, USA; ^4^ Bakar Computational Health Sciences Institute, University of California, San Francisco, San Francisco, CA, USA; ^5^ Graduate Program in Biological and Medical Informatics, University of California, San Francisco, San Francisco, CA, USA; ^6^ Department of Cognitive Science, University of California, San Diego, La Jolla, CA, USA; ^7^ Department of Neurosciences, University of California, San Diego, La Jolla, CA, USA; ^8^ Movement Disorders and Neuromodulation Center, Department of Neurology, Weill Institute for Neurosciences, University of California, San Francisco, San Francisco, CA, USA; ^9^ Department of Psychiatry, University of California, San Francisco, San Francisco, CA, USA; ^10^ Centre for Precision Psychiatry, Division of Mental Health and Addiction, Oslo University Hospital and Institute of Clinical Medicine, University of Oslo, Oslo, Oslo, Norway; ^11^ Center for Multimodal Imaging and Genetics, University of California, San Diego, La Jolla, CA, USA; ^12^ Division of Geriatrics, Gerontology & Palliative Care, Department of Medicine, University of California, San Diego, La Jolla, CA, USA; ^13^ Department of Radiology and Biomedical Imaging, University of California San Francisco, San Francisco, CA, USA; ^14^ Memory and Aging Center, Weill Institute for Neurosciences, University of California San Francisco, San Francisco, CA, USA; ^15^ Department of Neurology, Memory and Aging Center, University of California San Francisco, San Francisco, CA, USA; ^16^ Department of Pathology, University of California, San Francisco, San Francisco, CA, USA; ^17^ Global Brain Health Institute, University of California, San Francisco, San Francisco, CA, USA; ^18^ Memory and Aging Center, Weill Institute for Neurosciences, University of California, San Francisco, San Francisco, CA, USA; ^19^ Global Brain Health Institute (GBHI), University of California San Francisco (UCSF); & Trinity College Dublin, San Francisco, CA, USA

## Abstract

**Background:**

Dysregulation of the interferon (IFN) response is emerging as a major pathobiological contributor across multiple forms of neurodegeneration, but much less is known about how genetic variation in the IFN pathway modulates the course of neurodegenerative disease. IFN signaling represents a primary cellular mechanism for the antiviral response. Furthermore, both viral infection and vaccination are increasingly recognized for their roles in modifying neurodegenerative disease risk. We hypothesized that common variation in IFN‐stimulated gene *IFI44L*, which is enriched in IFN‐responsive immune‐cell subsets and strongly associated with vaccine response, would be associated with differences in clinical trajectories in neurodegenerative disease and normal aging.

**Method:**

We performed longitudinal analyses using linear mixed‐effects models on measures of clinical severity and cognitive impairment across four clinical diagnoses and two independent cohorts, including the University of California, San Francisco Memory and Aging Center (MAC) and the Alzheimer's Disease Neuroimaging Initiative (ADNI). Clinical measures included the Clinical Dementia Rating Scale Sum of Boxes (CDR‐SB) and the Mini‐Mental State Examination. *IFI44L* rs273259 genotype was measured by whole‐genome sequencing (MAC) and Illumina GWAS BeadChips (ADNI).

**Result:**

After controlling for baseline age, sex, education, *APOE* ε4 dosage, and self‐reported race, *IFI44L* genotype strongly associated with clinical trajectories in clinically normal (CN) individuals and those with mild cognitive impairment (MCI) in the ADNI cohort, with the rs273259 alternate (G) allele displaying a highly significant, dose‐dependent relationship with worse clinical trajectories (for CDR‐SB, in CN, *p* < 2 x 10^‐16^; in MCI, *p* = 1.1 x 10^‐6^; Figure 1A‐B). In the MAC cohort, the alternate allele was also associated with worse CDR‐SB trajectories in CN (*p* = 0.05), frontotemporal dementia (*p* = 3.9 x 10^‐10^; Figure 1C) and early‐onset AD (*p* = 3.3 x 10^‐3^; Figure 1D). Among subjects with neuropathologically defined frontotemporal lobar degeneration (FTLD) with tau or TDP‐43 proteinopathy, *IFI44L* genotype was associated with clinical trajectories in both subtypes (*p* = 3.8 x 10^‐5^, FTLD‐tau; *p* = 0.03, FTLD‐TDP).

**Conclusion:**

A common *IFI44L* variant is significantly associated with clinical trajectories in normal aging and in multiple forms of neurodegenerative disease, confirming an important role for IFN signaling in aging and neurodegeneration (Figure 2).